# Stress Mindset and Perceived Stress: No Momentary Links to Cognitive Functioning in Daily Life

**DOI:** 10.1093/geroni/igaf122.279

**Published:** 2025-12-31

**Authors:** Daisy Zavala, Amanda Miller, Martina Luchetti, Elizabeth Milad, Selin Karakose, Antonio Terracciano, Angelina R Sutin

**Affiliations:** Florida State University, Tallahassee, Florida, United States; Florida State University, Tallahassee, Florida, United States; Florida State University College of Medicine, Tallahassee, Florida, United States; Florida State University, Tallahassee, Florida, United States; Florida State University, Tallahassee, Florida, United States; Florida State University, Tallahassee, Florida, United States; Florida State University College of Medicine, Tallahassee, Florida, United States

## Abstract

Evidence suggests that individuals that are unable to adapt to daily stress are more likely to experience short-term cognitive failures. Yet, less is known about how a stress mindset affects perceptions of stress and cognition in daily life, and whether a stress mindset buffers against the negative effects of perceived stress on cognition. The present study leveraged a momentary ecological assessment (EMA) study to investigate the association between stress mindset, perceived stress, and cognitive functioning among middle-aged adults (40-70 years, N = 286). We hypothesized that a stress-is-enhancing mindset would predict lower momentary perceived stress, and that higher perceived stress would be associated with worse cognitive functioning in processing speed and working memory – as measured by the Dot Memory and Symbol Search task. Stress mindset was measured at baseline, whereas perceived stress and cognition were measured across an 8-day EMA protocol (3 assessments/day). Multilevel models that adjusted for temporal and contextual factors, age, sex, education, race/ethnicity, practice, and chronic stress, revealed no significant results. That is, stress mindset did not predict lower momentary perceived stress, nor were any momentary associations observed between perceived stress and cognitive functioning. However, people who were older reported less momentary perceived stress and those who had more years of education and chronic stress reported more momentary perceived stress. These findings suggest that further work is needed as theoretical and experimental work highlighting the benefits of a stress-is-enhancing mindset, may not fully translate to experiences of stress and cognition in daily life.

